# Special Issue “Targeting CAR T-Cell Therapy: Molecular Research and Its Future Implication”

**DOI:** 10.3390/ijms262210868

**Published:** 2025-11-09

**Authors:** Stella Bouziana

**Affiliations:** 1 Comprehensive Cancer Centre, School of Cancer and Pharmaceutical Medicine, King’s College London, London SE5 8AF, UK; styliani.bouziana@kcl.ac.uk or styliani.bouziana@nhs.net; 2 Department of Haematology, King’s College Hospital National Health Service (NHS) Foundation Trust, London SE5 9RS, UK

The emergence of chimeric antigen receptor (CAR) T-cell therapy represents a significant milestone in the cellular treatment of cancer. Rooted in advances in genetic engineering and immunology, CAR T-cell therapies have radically changed the treatment landscape in hematologic malignancies, even in patients with highly aggressive chemo-refractory or relapsed neoplasms [1]. By genetically engineering and redirecting autologous T-cells to recognize antigens on the tumour cells, this modality has managed to overcome tumour-related T-cell anergy and immune tolerance, leading to unprecedented remission rates in otherwise treatment-resistant cancers [2,3]. CAR T-cell therapies display great value as part of a personalized and targeted therapeutic strategy, opening novel avenues in cancer treatment. Currently, there are seven autologous CAR T-cell products approved by the Food and Drug Administration for use in the commercial setting to treat a variety of hematological malignancies, including both indolent and aggressive neoplasms [4].

Despite these clinical successes, CAR T-cell therapy remains constrained by several challenges. Among these is the risk of severe toxicities, including cytokine release syndrome and immune effector cell-associated neurotoxicity syndrome, which can be life-threatening and necessitate intensive supportive care [5]. In addition, long-term side effects such as prolonged cytopenias and infections, with the latter representing the main cause of non-relapse mortality, not only comprise a major burden for patients’ quality of life, but are also financially toxic for healthcare systems and insurance companies [6]. Moreover, the recently introduced safety signals of secondary malignancies post CAR T-cell therapy, including rare cases of secondary T-cell malignancies, represent a new concern for the scientific community [7]. These adverse events are often linked to the hyperactivation of immune pathways, prolonged subclinical inflammation, and immune dysregulation, underscoring the need for developing biomarkers and predictive or prognostic systems that can prognosticate toxicity and outcomes and guide patient selection.

Furthermore, despite good initial remission rates, the durability of these responses is frequently compromised by different mechanisms, including T-cell exhaustion and antigen escape. Exhaustion reflects a progressive loss of effector function, often driven by sustained antigen stimulation and inhibitory receptor expression, while antigen escape involves the downregulation or mutation of the targeted epitope, rendering CAR T cells ineffective. These phenomena highlight the dynamic interplay between engineered immune cells and tumour evolution, and they present formidable barriers to long-term disease control [8]. As a result, the majority of patients will eventually relapse, having a dismal outcome as effective treatment options are usually limited post CAR T-cell relapse, mainly restricted to treatments offered within the context of available clinical trials [9].

In response to these challenges, the field is rapidly advancing towards the molecular refinement of CAR T-cell constructs and novel modes of CAR expression. Strategies under investigation include the incorporation of co-stimulatory domains to enhance persistence, the use of gene-editing technologies to reduce immunogenicity, and the development of dual-targeting CARs to mitigate antigen escape. Additionally, synthetic biology approaches are enabling the design of switchable and logic-gated CARs that offer greater control over activation and specificity [10,11]. Furthermore, non-viral approaches are explored to express the CAR construct in targeted immune cells, including even in vivo manufacturing [12]. Moreover, along with T cells, alternative immune cells are also under investigation in terms of their safety and efficacy, including NK, iNK, iPSCs, macrophages, γδ T cells, and allogeneic T cells derived from healthy volunteers or umbilical cord blood [13,14]. These innovations aim not only to improve efficacy but also to reduce toxicity and broaden the therapeutic window. In addition, as technological advances continue to evolve, the integration of genomic, transcriptomic, and proteomic data will be essential to personalize treatment, predict outcomes, and minimize adverse effects.

Translational research is also exploring the extension of CAR T-cell therapy to solid tumours, a difficult-to-treat area given the multiple mechanisms of resistance that these tumours develop to immunotherapy. Efforts to overcome the immunosuppressive tumour microenvironment, enhance T-cell trafficking, and identify novel antigen targets are critical to this endeavour. Moreover, combination strategies involving checkpoint inhibitors and cytokine modulation are being evaluated to synergize with CAR T-cell activity [15]. Results of clinical trials investigating the potential of novel CAR constructs against solid tumours are eagerly anticipated to shed light on the therapeutics of these neoplasms.

This Special Issue committed to collecting the latest original and review articles covering molecular biological studies which investigate the strategies of mitigating CAR T-cell-related toxicities, the mechanisms of relapse post CAR T-cell therapy, and pathways for augmenting CAR T-cell efficacy and potency in malignant diseases. All submitted articles in this Special Issue underwent a rigorous peer review process, and ultimately five articles were published, including three original and two review articles. An overview of the contributed articles is provided below:

1. The results of the design and implementation of digital droplet PCR (ddPCR) assays to monitor CAR T-cell expansion were presented in an original article capturing the real-world experience of a clinical centre. The ddPCR assays showed sensitivity, precision, and reproducibility in monitoring longitudinal samples of multiple tissue types in patients treated with commercial CAR T-cell products for hematological B-malignancies, while the authors also correlated peak CAR T-cell expansion with clinical outcomes and adverse effects. (Wiedemann, G.; Bacher, U.; Joncourt, R.; Solly, F.; Widmer, C.C.; Zeerleder, S.; Novak, U.; Pabst, T.; Porret, N.A. A Comprehensive ddPCR Strategy for Sensitive and Reliable Monitoring of CAR-T Cell Kinetics in Clinical Applications. Int. J. Mol. Sci. 2024, 25, 8556. https://doi.org/10.3390/ijms25168556) [16].
2. Novel endogenous signalling molecule activating (ESMA) CAR structures, which eliminate the need for stimulatory signals within the CAR, were initially tested in vitro and then the lead candidate was investigated in vivo in a triple-negative breast cancer (TNBC) mouse model. The alternative ESMA CARs triggered robust cytotoxic activity and proliferation and reduced cytokine secretion and exhaustion markers in vitro against the TNBC cell line MDA-MB-231, while the lead candidate showed profound tumour infiltration, repression of tumour growth, and enhanced T-cell memory formation in a NSG MDA-MB-231 xenograft mouse model. (Ebbinghaus, M.; Wittich, K.; Bancher, B.; Lebedeva, V.; Appelshoffer, A.; Femel, J.; Helm, M.S.; Kollet, J.; Hardt, O.; Pfeifer, R. Endogenous Signaling Molecule Activating (ESMA) CARs: A Novel CAR Design Showing a Favorable Risk to Potency Ratio for the Treatment of Triple Negative Breast Cancer. Int. J. Mol. Sci. 2024, 25, 615. https://doi.org/10.3390/ijms25010615) [17].
3. The potency of a novel CAR construct targeting the stage-specific embryonic antigen 4 (SSEA-4), which is overexpressed in TNBC, was investigated in in vitro and in vivo conditions. In vitro conditions showed antigen-specific T-cell activation and the killing of SSEA-4-expressing target cells when different CAR contrasts containing alternative extracellular spacer domains were tested. Unexpectedly, the most bioactive CAR T-cell construct showed severe on-target/off-tumour toxicity with limited anti-tumour efficacy when injected into TNBC xenograft mice due to the expression of the SSEA-4 antigen in progenitor cells in the lung and bone marrow. (Pfeifer, R.; Al Rawashdeh, W.; Brauner, J.; Martinez-Osuna, M.; Lock, D.; Herbel, C.; Eckardt, D.; Assenmacher, M.; Bosio, A.; Hardt, O.T.; et al. Targeting Stage-Specific Embryonic Antigen 4 (SSEA-4) in Triple Negative Breast Cancer by CAR T Cells Results in Unexpected on Target/off Tumor Toxicities in Mice. Int. J. Mol. Sci. 2023, 24, 9184. https://doi.org/10.3390/ijms24119184) [18].
4. A review article presented the crucial role of beta2-adrenergic receptors (β2-ARs) in modulating the functioning of immune cells and controlling immunological responses against tumour cells. Exploring the emerging data of the immunosuppressive effects of β2-ARs on T cells could render β2-ARs as a targetable checkpoint in CAR T-cell therapies, which could further augment the potency of T cells. (Farooq, M.A.; Ajmal, I.; Hui, X.; Chen, Y.; Ren, Y.; Jiang, W. β2-Adrenergic Receptor Mediated Inhibition of T Cell Function and Its Implications for CAR-T Cell Therapy. Int. J. Mol. Sci. 2023, 24, 12837. https://doi.org/10.3390/ijms241612837) [19].
5. Strategies for reducing CAR T-cell-related toxicities and enhancing their efficacy in hematological malignancies were reviewed. Several novel approaches were presented in three different sections with the aim of summarizing current and emerging knowledge. Some of these strategies included modification of the CAR T cells by means of the use of gene-editing technologies, combination with other anti-tumour drugs and the development of next-generation CAR constructs. (Wang, H.; Tang, L.; Kong, Y.; Liu, W.; Zhu, X.; You, Y. Strategies for Reducing Toxicity and Enhancing Efficacy of Chimeric Antigen Receptor T Cell Therapy in Hematological Malignancies. Int. J. Mol. Sci. 2023, 24, 9115. https://doi.org/10.3390/ijms24119115) [20].

We hope that the cutting-edge research presented in this Special Issue will help to advance the understanding of CAR T-cell biology and inform its clinical translation. Continued interdisciplinary collaboration will be key to unlocking the full potential of this promising therapeutic frontier. Ultimately, the goal is to transform CAR T-cell therapy from a niche intervention into a broadly applicable, curative modality.
